# Ideational and conceptual apraxia by cerebral infarction in the left basal ganglia and right pons: a case report

**DOI:** 10.3389/fresc.2025.1723964

**Published:** 2026-01-14

**Authors:** Yuka Nakaya, Koji Hayashi, Asuka Suzuki, Mamiko Sato, Naoko Takaku, Toyoaki Miura, Hiromi Hayashi, Kouji Hayashi, Yasutaka Kobayashi

**Affiliations:** 1 Department of Rehabilitation Medicine, Fukui General Hospital, Fukui, Japan; 2 Graduate School of Health Science, Fukui Health Science University, Fukui, Japan; 3 Department of Neurology, University of Fukui Hospital, Fukui, Japan

**Keywords:** apraxia, basalganglia, case report, cerebral infarction, conceptual apraxia, ideational apraxia, pons

## Abstract

We report a rare and uncommon case of ideational apraxia (IA) and conceptual apraxia (CA) in a 70-year-old woman after concurrent cerebral infarctions of the left basal ganglia (BG) extending to the corona radiata and the right pons. The patient abruptly developed difficulty operating devices such as the car's gear shift and smartphone. The brain MRI on admission disclosed infarction in the area involving the left BG and right pons. During rehabilitation therapy, she exhibited features of IA and CA, including sequencing failures when using technological devices and a profound loss of functional knowledge, exemplified by an inability to recall the purpose and functions of an automated teller machine (ATM) despite prior proficiency. A testing error involved using the keyboard rather than the mouse to open a file. Behaviorally, she impulsively initiated actions and sometimes scheduled conflicting appointments. Neuropsychological testing showed preserved scores on the Mini-Mental State Examination (MMSE) and the Hasegawa Dementia Scale-Revised (HDS-R), but memory impairment was evident on the Rivermead Behavioural Memory Test (RBMT) and the Standard Verbal Paired Associates Learning Test (S-PA). The Behavioural Assessment of the Dysexecutive Syndrome (BADS) revealed marked dysexecutive symptoms, and the Clinical Assessment for Attention (CAT) indicated reduced auditory selective attention and diminished inhibitory control. Notably, the Standard Performance Test for Apraxia (SPTA) yielded normal results. Treated with antiplatelets then antihypertensives, she was discharged home four months later. Attention deficits and driving cessation persisted, but daily life was minimally affected. Although IA and CA are typically linked to left-hemisphere damage, particularly to the BG, the right pontine lesion may have contributed to the deficits, possibly through network disruption. This infarct pattern suggests a complex interplay in apraxia development and warrants further mechanistic study.

## Introduction

Ideational apraxia (IA) is defined as a high-order cognitive disturbance involving the conceptual understanding of object use and the proper sequencing of tasks, leading to difficulty performing complex, multistep actions despite preserved elemental motor and sensory functions (1–5). IA is fundamentally characterized by a failure to plan and execute learned action sequences (2). This deficit often includes a loss of knowledge about the object's purpose, historically referred to as “amnesia of usage” or a disruption in accessing semantic tool representations (1, 4, 5). This framework aligns with cognitive models of action control, such as the dual-systems theory proposed by Norman and Shallice, where routine action sequences are governed by automatic schema selection (contention scheduling, CS), and deficits like IA are often linked to a failure in these underlying selection mechanisms (5, 6).

Conceptual apraxia (CA), on the other hand, involves a more fundamental impairment in semantic knowledge of objects and actions, resulting in incorrect use and an inability to recognize the broader tool purpose (3, 7). CA is recognized as a more severe action-semantic deficit that frequently overlaps with or evolves from IA, reflecting a conceptual continuum of high-level praxis failure (3, 7).

Here, we report a rare and anatomically uncommon case of a patient presenting with both IA and CA following concurrent cerebral infarction in the left basal ganglia (BG) extending to the corona radiata and the right Pons.

## Case presentation

**Figure 1:**
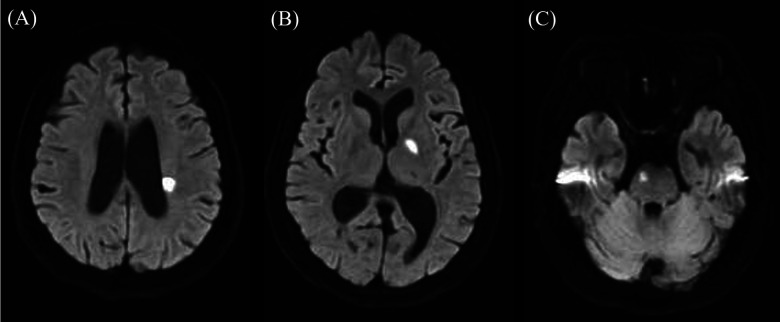
Diffusion-weighted brain magnetic resonance imaging (MRI) findings. **(A,B)** Diffusion-weighted imaging showing a hyperintense lesion extending about 20 mm from the left corona radiata to the basal ganglia. **(C)** Hyperintensity observed in the right pons. Image slice thickness: 5 mm.

**Figure 2:**
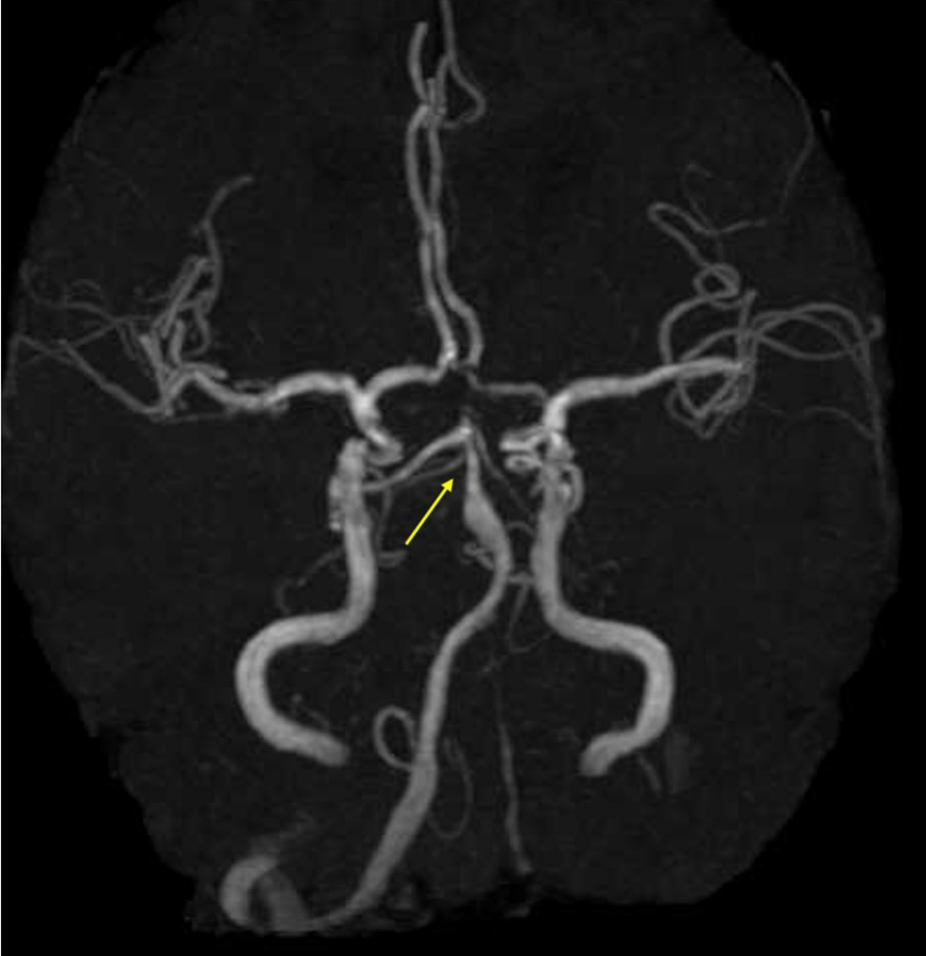
Brain magnetic resonance angiography (MRA) findings. MRA shows approximately 50% stenosis of the basilar artery and hypoplasia of the left vertebral artery.

A 70-year-old woman with hyperlipidemia suddenly developed difficulty operating machines. She could not operate the car's gear shift or rev the engine without pressing the throttle. Based on interviews with her and her family, as well as her medical records, she can work on the computer but cannot click the mouse or operate her smartphone. She was a native Japanese speaker and operates only within a monolingual environment. Since slurred speech was pointed out by her family, she visited the hospital together with her family the day after the onset. Vital signs showed a blood pressure of 210/93 mmHg and a heart rate of 64 beats per minute. Neurological examination revealed mild right facial nerve paralysis, bilateral motor impairment in the finger-nose-finger test, right-hand clumsiness during the pronation test, and hyperreflexia in both upper limbs. Limb paralysis, sensory disturbance, gait disturbance, or pathological reflexes were not observed. Blood tests showed elevated lactate dehydrogenase levels (Table 1). Brain magnetic resonance imaging (MRI) with diffusion-weighted imaging (DWI) revealed hyperintensities that were concordant with hypointensities on apparent diffusion coefficient (ADC) maps and subtle hyperintensities on T2-FLAIR sequences in the left BG extending to the corona radiata (4 slices, 20 mm) and in the right-central pons (Figure 1). Brain magnetic resonance angiography (MRA) demonstrated stenosis (>50%) of the basilar artery and hypoplasia of the left vertebral artery (Figure 2). Transthoracic echocardiography showed no valvular disease or intracardiac thrombus. Duplex ultrasonography showed bilateral carotid bulbs with a maximum plaque thickness of 3.8 mm, an estimated stenosis of approximately 30% without increased flow velocity. The Holter monitor did not detect atrial fibrillation.

**Table 1:** Blood test results on admission.

Inspection items	Result	Reference range
White blood cell count	47 × 10^2^/μl	(3,300–8,600)
Red blood cell count	454 × 10⁴/μl	(386–492 × 10⁴)
Hemoglobin	13.4 g/dL	(11.6–14.8)
Blood platelet	25.3 × 10⁴/μl	(15.8–34.8)
Blood urea nitrogen	12.2 mg/dL	(8.0–20.0)
Creatinine	0.54 mg/dL	(0.46–0.79)
Ammonia	64 μg/dL	(12–70)
Total bilirubin	0.7 mg/dL	(0.4–1.2)
Aspartate aminotransferase	20 U/L	(8–30)
Alanine aminotransferase	16 U/L	(7–23)
Alkaline phosphatase	96 U/L	(38–113)
Lactate dehydrogenase	237 U/L	(119–229)
γ-glutamyltransferase	18 U/L	(9–32)
Creatine phosphokinase	118 U/L	(41–153)
Amylase	83 U/L	(44–132)
Sodium	140 mmol/L	(138–145)
Potassium	3.8 mmol/L	(3.6–4.8)
Chlorine	104 mmol/L	(101–108)
C-reactive protein	0.03 mg/dL	(0.00–0.14)

Observations from rehabilitation sessions and the hospital ward revealed the following behavioral characteristics. The patient frequently initiated actions before instructions were fully given and exhibited impulsivity, such as exiting the room without checking for safety. Despite being informed of scheduled rehabilitation times in advance, the patient occasionally arranged conflicting appointments. Household tasks were performed smoothly and without difficulty.

However, difficulties were noted in operating electronic devices and machinery. Notably, the patient was unable to understand how to use a car's gear shift lever, which was also the chief complaint at the onset of cerebral infarction. The patient did not recall the function of an automated teller machine (ATM), including its basic purpose. According to her daughter, the patient had previously worked in accounting and had been proficient in using ATMs and banking systems. Nonetheless, she currently demonstrates a lack of understanding regarding ATM functions, recognizing only that money can be withdrawn but expressing uncertainty about the process (e.g., questioning whether inserting a card alone results in cash withdrawal). Furthermore, she appears to have forgotten that ATMs can be used for additional operations such as bank transfers. Similar difficulties were observed with computers and smartphones. When instructed to open a file on the desktop, the patient attempted to use the keyboard instead of the mouse. She was unable to name the functions of some keyboard keys and did not understand the role of the smartphone's home button.

Neuropsychological assessments are summarized in Table 2. While scores on the Hasegawa Dementia Scale-Revised (HDS-R) (21/30) and the Mini-Mental State Examination (MMSE) (25/30) remained within the preserved range, even when considering her educational history of 12 years (8). Significant impairments were observed in memory function, as evidenced by suboptimal performance on both the Standard Verbal Paired Associates Learning Test (S-PA) (9), a verbal memory test developed and standardized in Japan, and the Rivermead Behavioural Memory Test (RBMT). Additionally, the Clinical Assessment for Attention-revised (CAT-R), a standardized attention function assessment battery in Japan (10), revealed a selective profile of attention deficits consistent with diminished inhibitory control (Table 2, Supplementary Table 1). Specifically, the Auditory Detection subtest indicated deficits in auditory selective attention (Accuracy: 90%; Hit Rate: 37.2%; normative cutoff for Accuracy: 94%). In contrast, other subsets were preserved based on the 70s age group criteria. Executive dysfunction was further supported by the Behavioural Assessment of the Dysexecutive Syndrome (BADS), which revealed marked inefficiency and impaired executive processes (total profile score of 10/24; Table 2). The detailed scores of the six subtests confirmed pervasive deficits in planning, sequencing, problem-solving, and inhibitory control (See Supplementary Table for details). Additionally, the Standard Performance Test for Apraxia (SPTA), a standard test for higher motor disorders, primarily apraxia, in Japan, yielded normal results. She was treated with antiplatelets, followed by antihypertensives. Rehabilitation included conversation, memory, attention, executive, calculation training, and cognitive-behavioral therapy for movement agitation, planned in stages. After about four months, attention deficits remained, and driving was discontinued, but daily life was minimally affected, so she was discharged home. A follow-up assessment using the CAT demonstrated notable improvements in most domains (Supplementary Table 3), including auditory selective attention and processing speed. For instance, the Paced Auditory Serial Addition Test (PASAT) 2-second condition score improved significantly to 70% accuracy [compared to a normative mean of 49.7% (± 19.6) and a cutoff of 30%].

**Table 2:** Summary of neuropsychological assessment results.

Test name (battery)	Result (summary)	Below cutoff
Cognitive screening & general function		
Hasegawa dementia scale-revised (HDS-R)	21/30, preserved	
Mini-mental state examination (MMSE)	25/30, preserved^a^	
Frontal assessment battery (FAB)	15/18, preserved	
Raven's coloured progressive matrices (RCPM)	Within average range	
Kohs block design test	IQ 87.5	
Visual perception test for agnosia (VPTA)	Intact	
Executive function		
Behavioural assessment of the dysexecutive syndrome (BADS)	Marked inefficiency and impairment	Y
Trail making test (TMT) part A & B	Within normal limits	
Attention & inhibitory control		
Clinical assessment for attention (CAT)	Decline observed	Y
Memory & verbal learning		
Rivermead behavioural memory test (RBMT)	Deficits observed	Y
Standard verbal paired associates learning test (S-PA)	Impaired for related word pairs	Y
Praxis and language		
Standard performance test for apraxia (SPTA)	No abnormalities	
Kana retrieval task	Age-appropriate performance	
Standard language test of aphasia (SLTA)	Preserved; no aphasia	

Some neuropsychological tests are mainly used in Japan. Y; yes (below cutoff).

^a^ High education group's (>12 years educational history cutoff; 24/30 (8).

## Discussion

This case involves a monolingual patient who concurrent infarcts in the left corona radiata/BG and the right pons. Despite the absence of motor paralysis or core linguistic deficits (no aphasia on the SLTA), these lesions resulted in significant impairments in tool use and machinery operation.

A central interpretative challenge in this case is reconciling her normal results on SPTA with the clinical diagnosis of IA and CA. IA is defined as a disturbance in conceptual understanding of object use and task sequencing, characterized by failure to plan and execute learned action sequences (1–5). CA, distinct from IA, involves a more fundamental impairment in the semantic knowledge of objects and actions, historically termed “agnosia of usage” or semantic amnesia (3, 7, 11). Apraxia testing often evaluates two components: the ideational (conceptual plan) and the executive (movement execution) (11, 12). While the SPTA is a standardized tool for identifying various forms of apraxia, including IA and ideomotor (IMA) components, it primarily evaluates highly automated, routine sequences such as lighting a candle or placing a letter in an envelope (13). Therefore, SPTA assessment items were limited to relatively simple, automated, routine sequential operations and did not capture conceptual obstacles in more abstract, complex equipment operations that require higher-level planning, such as ATM and gear operations.

Instead, her diagnosis of IA/CA was strongly justified by a consistent pattern of ecologically valid errors related to complex devices (e.g., car's gear shift, ATM). For instance, she had difficulty operating modern devices, expressing uncertainty regarding required sequential steps (e.g., card insertion process or selecting transfers), despite prior proficiency. Her profound inability to recall basic functional knowledge, such as “not recalling ATM functions” or having “forgotten that ATMs can be used for additional operations like bank transfers,” pushed the diagnosis towards CA (3, 7). A classic “argument error” was also observed when she attempted to use the keyboard instead of the mouse to open a file (6). These high-level failures, not attributable to elemental motor or sensory deficits, confirm the impairment resides in higher-order cognitive processing (IA/CA) and conceptual storage of action, rather than in the motor execution domain evaluated by tests focused on ideomotor apraxia (11, 12).

The patient's errors in tool use provide insights into the underlying cognitive deficit. For example, attempting to use the keyboard instead of the mouse for file opening exemplifies an “argument error” (6). Cooper and Shallice's model posits that routine action selection relies on dynamic interaction between action schemas and object representations (5, 6). Misuse errors occur when an inappropriate object representation is incorrectly selected for an activated schema (6). This suggests a selective disruption in mapping functional goals to appropriate tools, distinct from general memory loss, aligning with IA from CS failure.

This functional description is critical given the patient's cognitive profile. Despite normal MMSE/HDS-R scores, significant memory impairment (RBMT, S-PA) indicated disrupted episodic memory and verbal learning. The S-PA deficit in paired word associations may reflect impaired acquisition/retrieval of action-semantic associations, contributing to conceptual difficulties. Grammatical processing impairment was unlikely, as SLTA confirmed no aphasia. CA, defined as semantic knowledge failure for objects/actions (e.g., ATM functions), signifies a deficit in accessing high-level lexical-semantic knowledge, not grammatical breakdown (11). Thus, S-PA/RBMT impairment reflects disrupted acquisition/retrieval of complex, action-specific semantic representations required for tool utilization.

Additionally, BADS revealed executive inefficiency, and CAT reported diminished inhibitory control. This suggests broader higher-order cognitive control impairment (supervisory attentional system; SAS dysfunction) beyond simple memory/attention deficits, critically undermining routine action integrity, especially combined with S-PA/RBMT difficulties. Per Norman and Shallice's dual control theory, executive/inhibitory deficits relate to SAS dysfunction, impairing willed control (5, 6). SAS impairment reduces top-down CS influence, leading to inappropriate schema selection (6), thereby exacerbating IA symptoms like argument errors and sequencing failures. The composite IA/CA presentation suggests a dual breakdown in action control, stemming from CS impairment (causing sequencing/argument errors) and SAS functional weakness (resulting in poor top-down monitoring/inhibition).

Notably, deficits were exacerbated with complex technological devices (e.g., ATM, smartphone) compared to simpler tasks. Their reliance on abstract, hierarchical action knowledge, involving arbitrary sequential steps and non-physical affordances, likely accounts for the profound deficits in high-level conceptual planning, while simpler manipulation schemas remained functional in household tasks.

The neuroanatomical basis of IA and CA involves distinct but interconnected regions. IA is primarily associated with damage in the left hemisphere, including the temporoparietal junction, inferior parietal lobule, and premotor and prefrontal cortices (1–3, 5). Parietal lesions impair object- and action-knowledge and sequencing, while injury to the BG—particularly the left BG—affects sequencing, movement control, response inhibition, and the temporal aspects of learned actions (2, 3). The BG's role in praxis extends to sequencing, fine-tuning movements, selecting motor programs, and supporting conceptual action knowledge via fronto-striatal circuits, as evidenced in Parkinson's disease and other disorders (1–3, 14). Though the exact nuclei within the BG and the specific white matter pathways remain indeterminate based on conventional MRI, the involvement of the left BG supports its role in core CS failure, manifesting as sequencing and argument errors during complex routines (1–3).

CA, alternatively, is linked mainly to damage in the left posterior hemisphere, especially the parietal lobe, and involves a fundamental loss of object and action semantics—often termed semantic amnesia or “agnosia of usage” (1, 7, 11). This condition involves a widespread neural network including premotor, prefrontal, temporal, and parietal regions associated with semantic memory (1, 14, 15).

Regarding the pontine lesion, literature is limited regarding direct links to apraxia, but recent studies show that pontine injury often causes executive dysfunction—affecting planning, initiation, organization, and self-monitoring—as well as impairments in working memory, inhibition, and attention (16, 17). These deficits likely result from diaschisis within the fronto–ponto–cerebellar–thalamic loop, where the pons acts as a key node, impairing hierarchical information processing necessary for higher cognition (16, 17). Evidence suggests that right-sided pontine strokes more frequently impact cognitive functions than left-sided, indicating potential hemispheric vulnerability (18).

In this case, the combination of a left BG infarct and right pontine stroke provides a plausible mechanistic explanation for the patient's complex IA and CA features. The left BG lesion might cause core CS failure, disrupting sequencing and response inhibition, leading to argument errors. Meanwhile, the right pontine infarct might impair higher-order control via disruption of the fronto–ponto–cerebellar–thalamic loop, affecting the SAS and thus top-down monitoring, inhibition, and goal maintenance. This dual pathology—core CS failure compounded by SAS dysfunction—may account for the severity and breadth of her apraxic and conceptual deficits. However, we acknowledge the limitation that specific basal ganglia nuclei or white matter pathways cannot be definitively identified solely through conventional MRI findings. Further research is needed to clarify the precise roles of these regions in IA and CA.

## Conclusion

This case highlights a patient presenting with IA and CA following concurrent left BG and right pontine infarcts. While IA and CA are primarily associated with left hemispheric damage, particularly BG, we acknowledge the anatomical limitation that the exact basal ganglia nuclei or the specific fronto-striatal white matter pathways within the corona radiata responsible for the hypothesized functional deficits cannot be definitively identified based solely on the conventional MRI findings. The right pontine lesion, known to cause significant executive and cognitive dysfunction via network disruption, may have contributed to the profound observed deficits. This unique combination of infarcts suggests a complex interplay in apraxia development, warranting further investigation into the precise underlying mechanisms.

## Data Availability

The original contributions presented in the study are included in the article/Supplementary Material, further inquiries can be directed to the corresponding author.
